# 

**Published:** 1883-03

**Authors:** 


ESTABLISHED IN 1855.

OLD SERIES. Vol. XX—No. 12. MARCH, 1883. NEW SERIES. Vol. II—No. 6.

### ATLANTA MEDICAL REGISTER.

(*ATLANTA MEDICAL AND SURGICAL JOURNAL.*)

EDITED BY

JAMES B. BAIRD, M.D.

H. H. DICKSON, PUBLISHER. TERMS, $2.50 A YEAR IN ADVANCE.

ATLANTA, GEORGIA:
*H. H. DICKSON, BOOK AND JOB PRINTER AND BOOKBINDER.*
1883.

